# Best Mode of Giving Ergot

**Published:** 1880-10

**Authors:** 


### EXCERPTA.

Best Mode of Giving Ergot.—Dr. A. Luton, Professor of Clinical Medicine in the school at Rheims, discusses this question quite fully, and strongly commends the hypodermic use of this drug whenever it is indicated. He is not in favor of any of the so-called ergotines of commerce, and prefers the simple alcoholic tincture of the Codex. He contends that the vehicle, alcohol, is no more irritating than those advised to dissolve ergotine, such as glycerine or chloroform, ether, etc., etc., which have also been injected under the skin. The pain produced by the alcoholic tincture is slight and transient, especially if the point selected for the injection has a thick layer cf cellular tissue, such as over the abdomen or toward the haunch. He has never observed an abscess to form after it has been used.

As to the dose, he had obtained as good results from one gram of the tincture (15.4 grains) injected, equivalent to twenty centigrams (3 grains) of the powder, as from the usual dose given by the mouth. Given by the mouth, ergot, like other fungi and highly nitrogenized bodies generally, must be partially digested or destroyed, hence we fail to get the full medicinal results of the dose, while, if it is given subcutaneously, we shall obtain its entire therapeutic power. Theoretically, we ought to reach definite results by giving ergot hypodermically, which we cannot expect when it is given by the mouth. Practically, we find this to be true. In two cases of hematuria treated by Prof. Luton, in the hotel Dieu of Rheims, no effects were produced by daily doses of ten grams of the tincture given by the mouth, while daily injections of one gram of the same tincture caused the symptoms to disappear in two or three days.—*Unlon Medet Scientif, du Nord-Est*, June 15, 1880.

